# Development of an Oncolytic Adenovirus with Enhanced Spread Ability through Repeated UV Irradiation and Cancer Selection

**DOI:** 10.3390/v8060167

**Published:** 2016-06-14

**Authors:** Stephen L. Wechman, Xiao-Mei Rao, Pei-Hsin Cheng, Jorge G. Gomez-Gutierrez, Kelly M. McMasters, H. Sam Zhou

**Affiliations:** 1 Department of Pharmacology and Toxicology, University of Louisville School of Medicine, Louisville, KY 40202, USA; slwech01@louisville.edu; 2 Department of Surgery, University of Louisville School of Medicine, Louisville, KY 40202, USA; jgguti01@louisville.edu; 3 James Graham Brown Cancer Center, University of Louisville School of Medicine, Louisville, KY 40202, USA; x0rao001@louisville.edu; 4 Department of Surgery, St. Jude Children’s Research Hospital, Memphis, TN 38105, USA; paisin.paisin@gmail.com; 5 Department of Microbiology and Immunology, University of Louisville School of Medicine, Louisville, KY 40202, USA

**Keywords:** lung cancer, adenovirus, E1b, autophagy, spread, oncolysis

## Abstract

Oncolytic adenoviruses (Ads) have been shown to be safe and have great potential for the treatment of solid tumors. However, the therapeutic efficacy of Ads is antagonized by limited spread within solid tumors. To develop Ads with enhanced spread, viral particles of an *E1*-wildtype Ad5 *dl*309 was repeatedly treated with UV type C irradiation and selected for the efficient replication and release from cancer cells. After 72 cycles of treatment and cancer selection, AdUV was isolated. This vector has displayed many favorable characteristics for oncolytic therapy. AdUV was shown to lyse cancer cells more effectively than both *E1*-deleted and *E1*-wildtype Ads. This enhanced cancer cell lysis appeared to be related to increased AdUV replication in and release from infected cancer cells. AdUV-treated A549 cells displayed greater expression of the autophagy marker LC3-II during oncolysis and formed larger viral plaques upon cancer cell monolayers, indicating increased virus spread among cancer cells. This study indicates the potential of this approach of irradiation of entire viral particles for the development of oncolytic viruses with designated therapeutic properties.

## 1. Introduction

Human adenoviruses (Ads) have been modified for selective replication in cancer cells, causing lysis [1]. Ads exhibit their favorable safety profile of oncolytic therapy because they only cause negligible flu-like symptoms and cannot integrate their genomes into the host cell chromosomes [2]. The therapeutic effects of oncolytic Ads are initiated from a small number of infected cancer cells from which the progeny viruses are released to further infect adjacent cancer cells within tumors [3,4]. However, preclinical and clinical studies have suggested that Ad spread is restricted in large tumors, limiting their therapeutic efficacy.

The Ad genome is composed of linear, double-stranded DNA of approximately 36 kb which can be divided into *early* (*E*) and *late* (*L*) genes based upon their expression across time [2,5]. The *L* genes encode structural proteins which package the viral DNA into the Ad virion during the final stages of replication. The *E* genes include *E1a, E1b, E2, E3*, and *E4* for regulation of viral replication. The proteins encoded by *E1a* are produced immediately after infection to modulate the cell cycle, recruit cellular proteins, and regulate the expression of cellular and viral genes [6]. The Ad *E1b* gene encodes two major polypeptides of 55,000 kDa (55K) and 19,000 kDa (19K). The expression of both *E1b55K* and *E1b19K* is required to transform rodent cells as shown following viral transduction and DNA transfection [7,8]. Both E1B55K and E1B19K proteins protect infected cells from E1A-induced stabilization of p53 and apoptosis [9]. E1B55K also enhances viral *E1a* expression [10] and is involved in the induction of the cyclin E gene which is required for Ad efficient replication [11,12,13,14,15,16]. The Ad E1B19K protein is a putative B-cell lymphoma 2 protein (Bcl-2) functional homolog and a strong inhibitor of apoptosis [17,18,19]. E1B19K prevents E1A-induced apoptosis by interfering with the actions of the pro-apoptotic proteins Bak and Bax [20]. The actions of these *E1b*-encoded proteins are therefore thought to maximize Ad replication.

The development of oncolytic Ads has focused primarily upon the genetic manipulation of *E1a* and *E1b*. At least two approaches have been applied for the development of oncolytic Ads to date. One approach is to delete or attenuate *E1b*, the expression of which is not essential for virus replication in cancer cells. For example, *dl*1520 (ONYX-015) is a gene-attenuated oncolytic virus with an 827-bp deletion and a nonsense point mutation in the *E1b* region that generates a premature stop codon to prevent the complete translation of the E1B55K protein [7,21,22]. Ad *dl*1520 has shown promising oncolytic efficacy in preclinical and clinical trials. Several hundred cancer patients were treated with *dl*1520 via various routes of administration in phase I and phase II clinical trials [23]. However, during a phase III clinical trial, when *dl*1520 treatment was combined with chemotherapy to treat head and neck squamous cell carcinoma patients, the trial was suspended due to the limited therapeutic effects [24]. Another approach to develop cancer selective Ads is to transcriptionally regulate the expression of essential viral genes with tumor-specific promoters to restrict the expression of viral regulatory genes, such as *E1a*, within tumors rather than normal tissue [4,25]. We recently developed Ad-cycE, in which *E1a* is regulated by the human cyclin E promoter [15,16,26]. Cyclin E overexpression has been observed in more than 90% of lung, liver, and gastrointestinal cancers, and in more than 80% of glioma/blastoma, bone, and breast cancers [27]. Furthermore, we observed that Ad infection further stimulated the activity of the cyclin E promoter, augmenting the oncolytic efficacy of Ad-cycE [11,12]. Ad-cycE can selectively replicate in a diverse range of cancer cells [26] and significantly repressed tumor growth, prolonging the survival of xenograft tumor bearing mice [15]. Other oncolytic Ads constructed with cancer-selective promoters, such as OBP-301 (Telomelysin) driven by human telomerase reverse transcriptase (hTERT) promoter [28] and CV706 driven by prostate-specific antigen (PSA) promoter [29], have progressed to human clinical trials.

Although Ads with *E1b* deletions and/or *E1a* regulated by cancer selective promoters have achieved some success in human clinical trials, the efficacy of oncolytic Ad virotherapy overall has been disappointing [22,24,30,31,32]. We previously reported that oncolytic Ads could efficiently inhibit the growth of small size tumors after intratumoral injection, but they were unable to repress the growth of large tumors [32]. This phenomenon was also observed during clinical studies as the direct injection of *dl*1520 did not inhibit the growth of larger tumors in patients [22,31]. It is likely that the restricted virus spread in large tumors is a major hurdle limiting the efficacy of virotherapy [33]. Thus, many extensive studies have suggested that the genetic manipulation of *E1a* and *E1b* was not sufficient to overcome this barrier. Therefore, it is necessary to explore new approaches for the development of more effective oncolytic Ads.

In this study, we treated the *E1*-wildtype Ad5 *dl*309 (Ad5) with UV irradiation, followed by selection in cancer cells. These treatments were repeated for total 72 cycles to select for Ads which can efficiently release from cancer cells. This approach targeted the entire genome rather than focusing exclusively upon the E1 region. Using this approach, we have isolated the oncolytic virus AdUV which displays the properties of enhanced oncolytic replication and spread *in vitro*.

## 2. Materials and Methods

### 2.1. Cell Lines and Culture Conditions

HEK293 (ATCC no. CRL-1573) human embryonic kidney cell, MRC5 (ATTCC no. CCL-171) human non-cancerous fibroblasts, HBEC (ATTCC no. CRL-4051) human non-cancerous epithelial cells, A549 (ATCC no. CCL-185) human lung carcinoma, H1299 (ATCC no. CRL-5803) metastatic human lung carcinoma of the lymph node, H441 (ATCC no. HTB-174) human lung papillary adenocarcinoma, MCF-7 (ATCC no. HTB-22) human breast adenocarcinomas, and Saos-2 human bone osteosarcoma (ATTCC no. HTB-85) cell lines were from the American Type Culture Collection (Rockville, MD, USA). A549, MCF-7, MRC5, and HEK293 cells were maintained in DMEM. H1299 and H441 cells were cultured in RPMI 1640 medium. Saos-2 cells were maintained in McCoy’s 5A media. HBEC cells were maintained in keratinocyte serum-free medium containing bovine pituitary extract and recombinant epidermal growth factor (Invitrogen, Waltham, MA, USA). All cell culture media was supplemented with 10% fetal bovine serum, 5% l-glutamine, and 5% penicillin/streptomycin (100 U/mL) unless otherwise indicated. All cells were cultured and maintained in humidified 5% CO_2_ incubators at 37 °C. All other cell culture reagents were obtained from VWR (VWR, Radnor, PA, USA) unless otherwise indicated.

### 2.2. Adenoviral Vectors

Ad5 *dl309* is *E1*-wildtype virus [34] and was used as a non-selective Ad control. Adhz60 with a deletion of the entire *E1b* gene was used as a cancer selective Ad control [35]. AdGFP is an Ad vector with the entire E1 gene (*E1a* and *E1b*) deleted, expressing GFP driven by the CMV promoter. AdGFP, as a negative control, does not replicate nor induce cytopathic effects (CPE). All Ads used in this study are based on the Ad5 backbone sequence.

**Figure 1:**
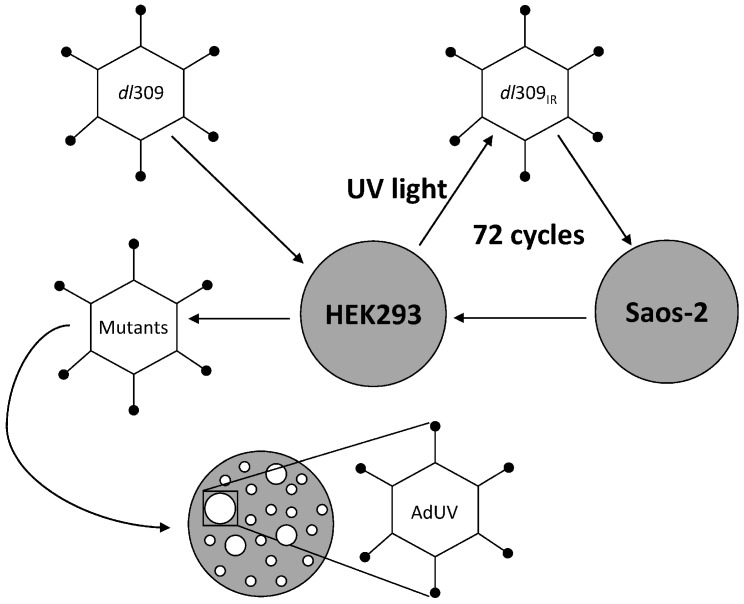
The repeated UV irradiation and cancer selection of AdUV. Ad5 *dl*309 was initially amplified in HEK293 cells prior to UV-irradiation. Irradiated viruses (*dl*309_IR_) were then selected in Saos-2 cancer cells which limit adenovirus replication. Viruses were then harvested from Saos-2 cells and amplified in HEK293 cells before subsequent cycles of UV irradiation. After 72 cycles of UV irradiation and cancer selection, AdUV was isolated by plaque purification from a large plaque formed upon Saos-2 cells.

To select Ads with increased spread, Ad5 *dl*309 was treated with UV irradiation for 5 min, inactivating approximately 90% of virus particles. The UV-light source was a germicidal lamp, USEG30T3 (Sylvania, Danvers, MA, USA), fitted with a 30 watt G30T8 UV-light bulb (Philips, Amsterdam, The Netherlands), which produces UV type-C irradiation. UV irradiation was measured with a model 25X UVX radiometer (Fisher Scientific, Pittsburgh, PA, USA). Using these conditions, 213 μW/cm^2^ UV type-C was produced, generating a total UV dose of 639 J/m^2^ after 5 min exposure. Irradiated viruses were then used to infect Saos-2 cancer cells, in which Ads cannot efficiently replicate [12]. Four hours after infection, virus particles in medium were removed by washing the cells with fresh media. Medium from infected monolayer cultures was collected at the first sign of CPE, generally at 24 h after infection, to harvest viruses that might replicate more efficiently in and release from the infected cancer cells. The harvested viruses were then amplified in 293 cells before being used for another cycle of treatment. These treatments were repeated for total 72 cycles and AdUV, that formed a large plaque on Saos-2 cells, was isolated (Figure 1).

### 2.3. Virus Titration and Release

Virus titer was determined using the TCID50 method as described previously [14]. HEK293 cells were seeded overnight onto 96-well plates at a density of 1 × 10^3^ cells per well and infected with virus samples serially diluted tenfold. The presence or absence of CPE in HEK293 cells was then recorded after a minimum of 7 days to calculate the virus titer.

To determine the release kinetics of AdUV, A549 cells were seeded onto 12-well plates at a density of 1 × 10^5^ cells per well. A549 cells were then infected with Ad5, Adhz60 or AdUV at an MOI of 1. Samples were collected at 6, 24, 36, 48, and 72 h after infection. The cell and cell culture media were separated by centrifugation at 2000 RPM (350 RCF) at 4 °C for 5 min using a micromax RF refrigerated microcentrifuge equipped with an IEC 851 rotor (Thermo Fisher Scientific, Waltham, MA, USA). Cell pellets were then resuspended in 1 mL of sterile PBS and subjected to 3 freeze-thaw cycles to release viruses contained within cells prior to titration. Cell free media samples were titered to detect for viruses released from cells. For studies focusing upon virus titer in the media alone, cells were infected at 1 MOI and media samples (20 μL) were collected daily for five days.

### 2.4. Western Blot Analysis

A549 cells were seeded onto 60 mm dishes overnight at a density of 6 × 10^5^ cells per dish. Cells were collected and centrifuged at 1500 RPM (453 RCF) at 4 °C for 5 min using an eppendorf 15 amp 5810 R refrigerated centrifuge equipped with a A-4-62 rotor (Eppendorf, Hamburg, Germany). The cell pellets were then washed with PBS prior to lysis with Radio immuno precipitation assay (RIPA) buffer containing 50 mM Tris-HCl, 150 mM NaCl, 1% NP-40, 0.5% sodium deoxycholate, and 0.1% sodium docecyl sulfate (SDS) with a protease inhibitor (PI) cocktail containing 4-(2-aminoethyl)-benzenesulfonyl fluoride (AEBSF), pepstatin A, trans-epoxysuccinyl-l-leucylamido-(4-guanidino)butane (E-64), bestatin, leupeptin, and aprotinin (10 mL/10^6^ cells; Sigma, St. Louis, MO, USA). The cell lysates were then incubated on ice in PI containing RIPA buffer for 30 min and homogenized every 10 min using a vortex-genie 2 (Scientific Industries, Bohemia, NY, USA). These lysates were then centrifuged at 14,500 RPM (196,000 RCF) at 4 °C for 10 min using a Micromax RF microcentrifuge equipped with an IEC 851 rotor to pellet cell debris formed during lysate preparation (Thermo Fisher Scientific). The pellet was discarded and the supernatant was stored at −80 °C for further experimentation. Protein concentrations were determined using the Pierce BCA protein assay kit according to the manufacturer’s instructions (Thermo Fisher Scientific). Equal amounts of cellular protein were resolved by electrophoresis through 8% (E1A, hexon, penton, protein V, protein VI, protein VII) or 12% (LC3-I and LC3-II) SDS-polyacrylamide gels prior to transfer to methanol activated PVDF membranes (GE healthcare, Little Chalfont, UK) using a semi-dry transfer apparatus (BIO-RAD, Hercules, CA, USA). Membranes were then blocked using 5% nonfat milk prepared in TBST for 1 h at room temperature. To detect protein expression, membranes were incubated with the following primary antibodies: rabbit-anti-adenovirus type 5 polyclonal antibody (1:10,000; abcam, Cambridge, UK), rabbit-anti-human LC3 monoclonal antibody (1:3000; Novus Biologicals, Littleton, CO, USA), rabbit-anti-human actin (1:2000; Sigma) or mouse-anti-adenovirus E1A antibody (1:1000; BD Pharmagen, San Jose, CA, USA) at 4 °C overnight on a lab-line thermal rocker (Thermo Fisher Scientific). Primary antibody binding was detected by the incubation with the horseradish peroxidase (HRP) linked anti-mouse or anti-rabbit immunoglobulin (Ig) diluted 1:5000 for 1 h at room temperature (Amersham, Piscataway, NJ, USA). All antibodies were diluted in TBST. Enhanced chemiluminescence (ECL) reagents were used to detect HRP-linked secondary antibody binding according to the manufacturer’s instructions (Amersham).

### 2.5. Plaque Formation Assay

A549 cells were seeded onto 6-well plates at a density of 6 × 10^5^ cells per well. Cells were then infected with Ad5, Adhz60 and AdUV at an MOI of 0.01. After 6 h, the cell culture medium was removed and replaced with 5 mL of DMEM 1% agarose solution per well. Images were taken six days post-infection with an EVOS FL microscope (Life Technologies, Carlsbad, CA, USA) at 4× total magnification. The pixels contained within ten representative plaques per Ad5, Adhz60, and AdUV treatment groups were quantified with the area density tool using Gel-pro analyzer 4.0 software according to the manufacturer’s tutorial (Media Cybernetics, Rockville, MD, USA). The total number of pixels for each plaque was converted to millimeters (mm) by dividing the number of pixels per plaque by the number of pixels per 1 mm^2^ (261,100 pixels) or squared pixel length of the 1000 micrometer (μm) scale bar (510 pixels).

### 2.6. Quantification of Band Intensity

Band intensities were quantified by Gel-pro analyzer 4.0 software in accordance with the manufacturer’s tutorial (Media Cybernetics). Densitometric values were expressed as the absolute integrated optical density (IOD) of each band normalized to actin expression as described previously [26]. Mock-treated cells were represented as 1-fold LC3-II/LC3-I expression for each experiment.

### 2.7. Cytotoxicity Assay

Cells were seeded overnight at a density of 2 × 10^4^ (H1299), 2.5 × 10^4^ (MRC5), 3 × 10^4^ (HBEC), 3 × 10^4^ (A549), 4 × 10^4^ (MCF-7), or 6 × 10^4^ (H441) cells per well onto 24-well plates. Cytotoxicity was assessed by crystal violet staining [36]. Suspended cells were removed by aspiration, the remaining adherent cells were then fixed with 3.7% formaldehyde for 20 min at room temperature. The excess formaldehyde was washed with PBS; the cells were then stained using 1% crystal violet at room temperature for 30 min. Excess crystal violet was washed away with water. Plates were then scanned using an HP Scanjet 4070 scanner (HP, Palo Alto, CA, USA). The remaining crystal violet was then solubilized with a 2% sodium dodecyl sulfate (SDS) solution and the sample absorbances were measured at 590 nm using a Synergy HT Multi-Mode Microplate Reader (Bio-Tek, Winooski, VT, USA). The absorbance (OD) values of each treatment were then normalized to mock-treated cells converting each sample OD into the percent (%) of cell viability according to the formula, cell viability % = (OD of treated cells/OD of mock-treated cells) × 100%. A549 cells displaying cytopathic effects were photographed at 200× magnification using an Olympus BX53 microscope (Olympus, Center Valley, PA, USA).

### 2.8. Statistical Analysis

All experiments were repeated at least three times. Quantification of results was reported as means of three independent experiments plus or minus (±) the standard deviation. The Pearson correlation coefficient (r) was used to evaluate the accuracy of equations used to calculate sample protein concentrations with the Pierce BCA protein assay kit. Statistical significance was assessed using analysis of variance analysis (ANOVA) for parametric and the Kruskal-Wallis test for non-parametric data. Multiple comparisons of ANOVA tests were corrected using Bonferroni’s method. Multiple comparisons of Kruskal-Wallis one-way analyses were corrected by Dunn’s test. All statistical tests were conducted using GraphPad PRISM 6 software (Microsoft, Redmond, WA, USA).

## 3. Results

### 3.1. AdUV Displays More Efficient Release and Cancer Cell Lysis

**Figure 2:**
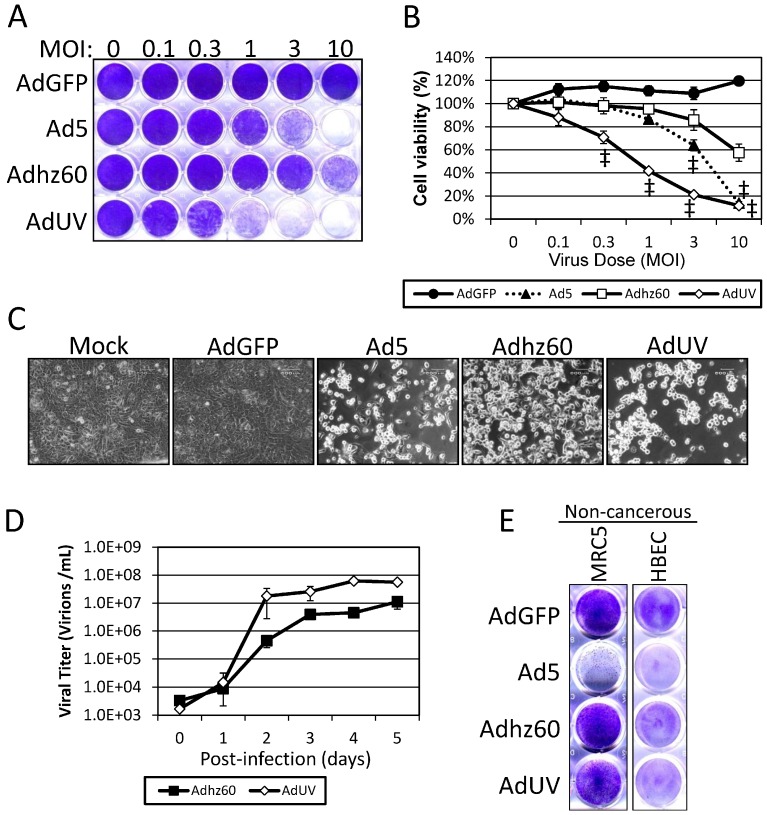
AdUV is released from and lyses A549 cells more efficiently than Adhz60. (**A**) A549 cells stained with crystal violet after five days’ treatment with the indicated Ads and MOIs; (**B**) Quantification of A549 cells stained with crystal violet expressed at the percent (%) viability; (**C**) Cytopathic effects (CPE) in A549 cells treated at an MOI of 1 for the indicated Ads were photographed at 200× total magnification five days post-infection; (**D**) A549 cells were infected with at an MOI of one with Adhz60 and AdUV and media samples were collected daily until day 5. Ad titer was determined via the TCID50 method using HEK293 cells. Day zero represents samples collected at 6 h post-infection; (**E**) Crystal violet staining of non-cancerous lung fibroblasts (MRC5) and epithelial (HBEC) cell lines treated at an MOI of 10 for three days. Quantified data are shown ± the standard deviation (SD) of three independent replicates. Statistical significance was assessed via two-way ANOVA relative to Adhz60 treated cells with multiple comparisons corrected for by Bonferroni’s method. ^‡^ indicates *p*-value > 0.001.

AdUV was generated as illustrated in Figure 1. Briefly, AdUV was isolated from a pool of Ad5 *dl*309 viral particles that were repeatedly treated with UV irradiation and selected in cancer cells for 72 cycles. To evaluate the potency of AdUV upon lung cancer cells, A549 cells were infected with the increasing MOIs of AdUV, AdGFP, Ad5, or Adhz60 for five days (Figure 2A). The differences among treatment groups were greatest at 3 MOI; these cancer cell viabilities were 20%, 60%, and 90% after treatment with AdUV, Ad5, or Adhz60, respectively (Figure 2B). Morphologically, AdUV induced greater cell rounding, consistent with cytopathic effects (CPE) relative to A549 cells treated with Adhz60, Ad5 (Figure 2C). These data indicate that AdUV efficiently destroyed A549 human lung cancer cells.

The enhanced lysis of A549 cells by AdUV prompted additional studies to determine whether this virus may release from cancer cells more efficiently. To follow the release kinetics of AdUV from cancer cells, A549 cells were infected with AdUV and Adhz60. Media samples were collected at 0, 1, 2, 3, 4, and 5 days post-infection to observe the release virus particles into culture media (Figure 2D). The titers for both viruses within the cell culture media increased with time; however, AdUV was released into the cell culture media more efficiently than Adhz60. At day 2, AdUV titer (3 × 10^7^) was 40 fold greater than Adhz60 (7.4 × 10^5^) in the cell culture media (Figure 2D). To evaluate the cancer selectivity of AdUV, MRC5 and HBEC non-cancerous cells were infected with AdUV, the cancer non-selective Ad5, and the cancer selective Adhz60 and AdUV at an MOI of 10. These data showed that AdUV lyse MRC5 and HBEC non-cancerous cells less efficiently than Ad5, but more than Adhz60, indicating the partial cancer selectivity of AdUV (Figure 2E).

**Figure 3:**
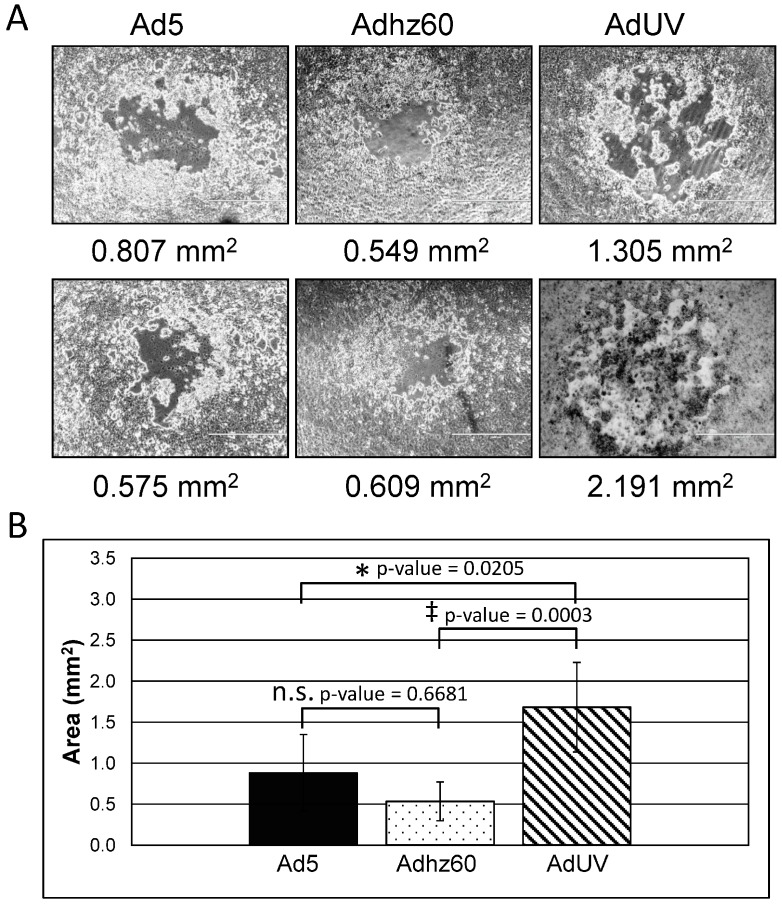
AdUV spread in A549 cells. (**A**) Two representative plaques of Ad5, Adhz60 and AdUV treated A549 cells are shown at 4× total magnification; (**B**) The number of pixels contained within each plaque was calculated by dividing by the number of pixels contained within each plaque by the number of pixels per 1 mm^2^ to determine the size of each plaque in millimeters (mm^2^). These data were then plotted as the average ± the standard deviation of ten plaques (SD). Statistical significance was assessed using the Kruskal-Wallis test. Adjusted *p*-values were reported for multiple comparisons via Dunn’s test. n.s. indicates *p*-value > 0.05, * indicates *p*-value < 0.05, ^‡^ indicates *p*-value < 0.001.

To verify the enhanced release of AdUV, the plaque assay was conducted. The plaque assay is based on the ability of a single infectious viral unit to replicate in cell and release a large number of progeny that can further infect neighboring cancer cells, finally forming a macroscopic area of cytopathology, or plaque, on a monolayer of cultured cells. Viruses which can efficiently replicate and release a greater number of viral particles will, therefore, form larger plaques. In this study, nearly confluent (70%) A549 cells were infected with Ad5, Adhz60, or AdUV. Plaque formation was observed four to five days post-infection. At day six post-infection, at least 50 plaques formed by each of the viruses were studied. The average size of plaques formed by AdUV, Ad5, and Adhz60 were 1.680, 0.883, 0.534 mm^2^, respectively; AdUV plaques were 314% larger than plaques formed by Adhz60 and 190% larger than those of Ad5 (Figure 3A,B; two representative plaques per treatment are shown). These differences were shown to be statistically significant. These data indicate that AdUV selected from the pool of mutated viruses releases and spreads more effectively than Ad5 and Adhz60.

### 3.2. Improved AdUV Replication in Cancer Cells

**Figure 4:**
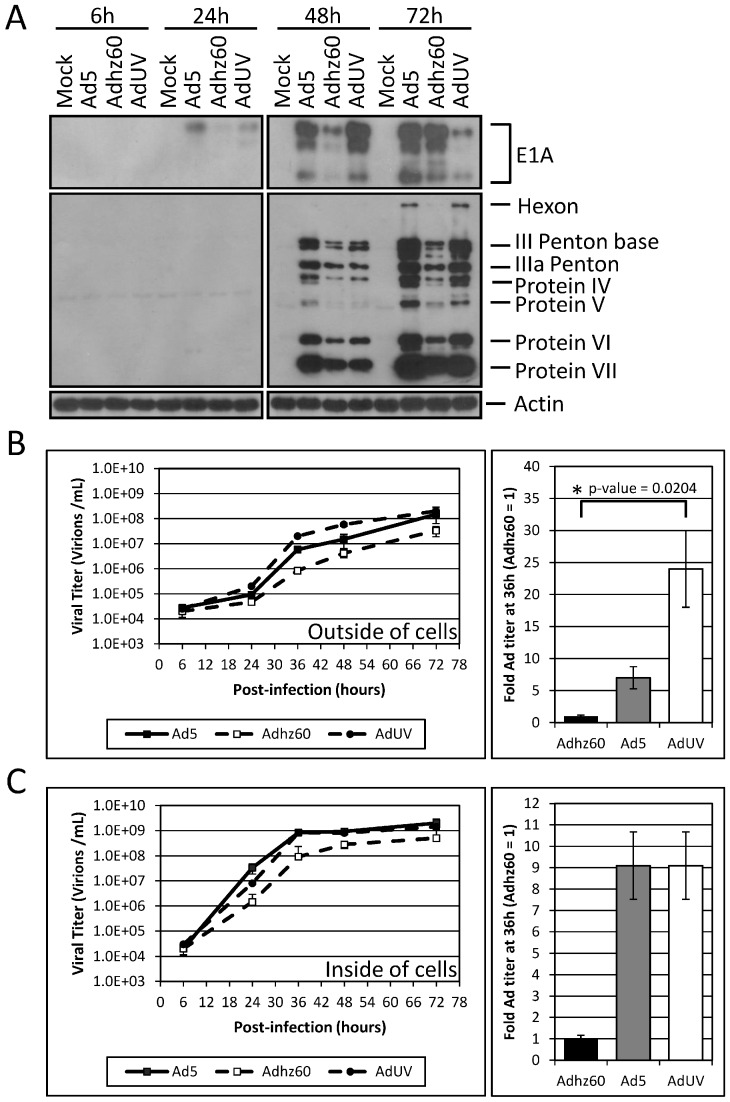
AdUV virus replication in A549 lung cancer cells. (**A**) The expression of the Ad E1A and late genes were observed in A549 cells treated with the indicated Ads at an MOI of 1 following 6 h, 24 h, 48 h, and 72 h infection; (**B**) Virus titer outside of A549 cells treated with the indicated Ads at an MOI of 1. Samples were collected at the indicated time-points. At 36 h, the number of virions of released in the cell culture media was seven-fold for Ad5 and 24-fold for AdUV when normalized to Adhz60 titer; (**C**) Virus titer inside of infected A549 cells treated with the indicated Ads at an MOI of 1. Samples were collected at the indicated time-points. At 36 h, the titers of AdUV and Ad5 inside of cells were nine-fold of Adhz60. Data is presented as the average titer ± the standard deviation (SD) of three independent replicates. Significance was determined via the Kruskal-Wallis test. Adjusted *p*-values were reported for multiple comparisons via Dunn’s test. h indicates hours, * indicates *p*-value < 0.05.

To investigate whether the ability of AdUV to form large plaques may be also related to increased virus replication in cancer cells, we studied the production of viral early (regulative) and late (structural) proteins in A549 cells. Adhz60 expressed much less E1A than the *E1b*-wildtype Ad5 in A549 cells at 48 h post-infection (Figure 4A); this is because the deletion of *E1b* in the vector resulted in repressed *E1a* expression [10]. At 48 h, the viral E1A production by AdUV peaked, even higher than that of Ad5. At 72 h, the expression of E1A by AdUV decreased rapidly, while Adhz60 increased (Figure 4A). Following the rapid expression of the viral early E1A proteins, AdUV late protein production also increased to significantly greater levels than Adhz60 at 72 h, to a level similar as Ad5 (Figure 4A).

To further study the oncolytic replication and release of AdUV, A549 cells were infected with AdUV, Ad5, or Adhz60 at an MOI of 1. In this experiment, the cell culture media and total cells were collected together at 6, 24, 36, 48, and 72 h post-infection. The cells and culture media were separated via centrifugation to determine the titers of viruses within cells and viruses released into the culture media. The titers of AdUV released in the media was 5 × 10^6^ at 36 h which was greater than both Ad5 (1.5 × 10^6^) and Adhz60 (1.5 × 10^5^; Figure 4B, left). When these data were normalized to Adhz60, AdUV titer was 24-fold higher than that of Adhz60, while Ad5 titer was seven-fold of Adhz60 (Figure 4B, right). The viral titers of AdUV and Ad5 within cancer cells were similar; the titers of both AdUV and Ad5 were consistently 5–10-fold greater than Adhz60 from 24 to 72 h and were nine-fold greater than Adhz60 at 36 h (Figure 4C). These results indicated that improved replication and rapid release of AdUV supports its ability to form large plaques and efficiently lyse cancer cells.

### 3.3. AdUV Induces Greater Autophagy

**Figure 5:**
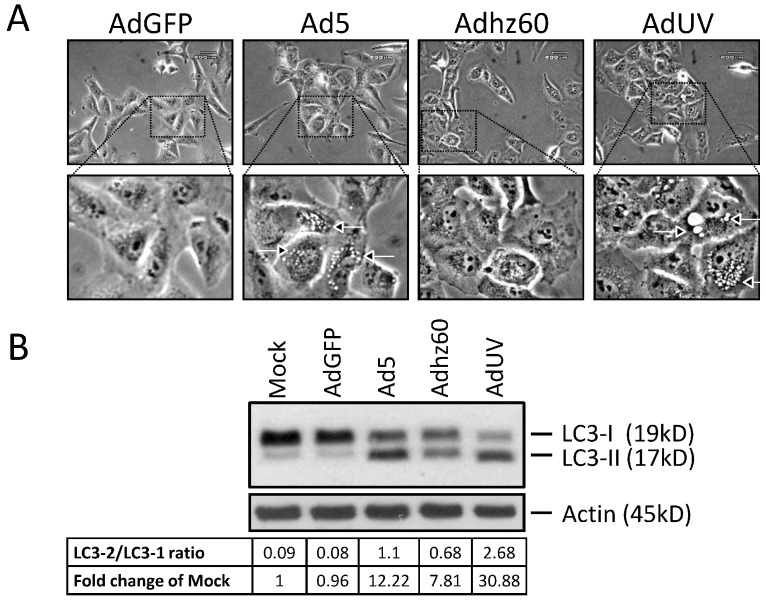
AdUV induces greater autophagy in A549 lung cancer cells. (**A**) Pictures of A549 cells (400× total magnifications) treated with the indicated Ads at an MOI of 1 for three days. Boxes indicate the magnified (~9.3× total magnifications) to emphasize the presence or absence of vesicle formation in the cytoplasm. White bordered arrows were used to indicate these vesicles; (**B**) A549 cells were treated with the indicated Ads at an MOI of 1 for five days and observed for LC3 expression by Western blot analysis. Densitometric analysis of LC3-II to LC3-I and actin were performed using Gel-pro analyzer 4.0 software. LC3 expression was normalized to actin expression.

We have previously shown that Ads induce autophagy to aid virus replication [37]. Autophagy leads to the sequestration of organelles and long lived proteins into autophagosomes, which then fuse with the lysosomes forming the autophagolysosome to recycle cellular components [38]. During autophagolysosome formation, microtubule-associated protein 1 light chain 3 (LC3) matures from LC3-I (18 kDa) to LC3-II (16 kDa) [39]. Therefore, the LC3-II/LC3-I protein ratio can be used to estimate the abundance of autophagosomes and indicate the relative levels of autophagy induction [40,41]. To study autophagy induction by AdUV, A549 cells were treated with AdGFP, Ad5, Adhz60, or AdUV at an MOI of 1. After three days, large vesicles were observed in the cytoplasm of A549 cells treated with Ad5 and AdUV, but not with AdGFP or Adhz60 (Figure 5A, arrowheads). The presence of such vesicles is a morphological marker of autophagy, prior to the complete lysis of A549 cells. Autophagy induction was evaluated further via the observation of LC3 expression by Western blot analysis. Relative to mock, the LC3-II/LC3-I expression ratio increased to 30.9, 12.2, and 7.8 fold for AdUV, Ad5, and Adhz60, respectively (Figure 5B). AdUV treatment displayed the greatest LC3-II/LC3-I expression ratio. These data indicate that AdUV induced autophagy much more effectively than both Ad5 and Adhz60 in A549 cells.

### 3.4. AdUV Kills a Variety of Cancer Cell Types

**Figure 6:**
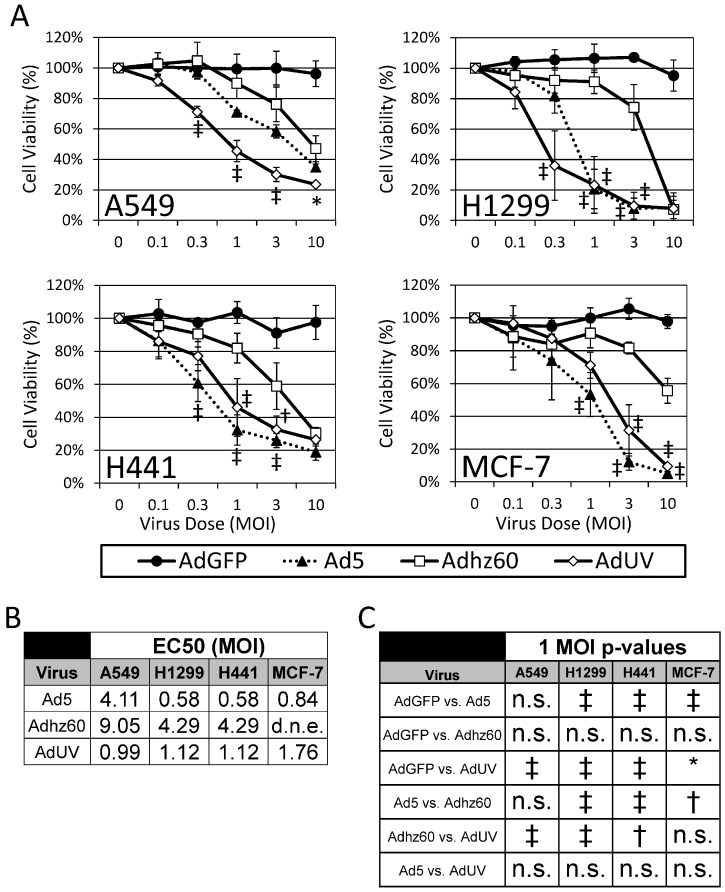
AdUV kills multiple cancer cell lines more effectively than Adhz60 *in vitro*. (**A**) A549, H1299, H441 lung cancer cells and MCF-7 breast cancer cells were treated with the indicated MOIs and Ads for five days prior to staining with crystal violet. The amount of crystal violet staining was quantified and expressed as the percent (%) viability of treated cells relative to non-treated cells ± the standard deviation of three independent experiments; (**B**) EC50 values were determined using GraphPad PRISM software; (**C**) Two-way ANOVA with multiple comparisons were conducted between all Ad treatments across all cancer cell lines at an MOI of 1. Adjusted p-values were reported for multiple comparisons via Bonferroni’s method. d.n.e. indicates the EC50 does not exist, * Indicates a *p*-value < 0.05, ^†^ indicates a *p*-value < 0.01, ^‡^ indicates a *p*-value < 0.001.

To study the effect of AdUV upon different cancer cells, A549, H1299, and H441 lung cancer cells, as well as MCF-7 breast cancer cells, were treated with increasing MOIs of AdGFP, Ad5, Adhz60, or AdUV for five days. Cell viability was then assessed via crystal violet staining and normalized to mock-treated cells (Figure 6A). AdGFP treatment did not alter cell viability while Ad5 efficiently lysed all cancer cell lines tested. AdUV lysed A549 cells significantly more efficiently than both Ad5 (*p*-value = 0.005) and Adhz60 (*p*-value = 0.0002) at 0.3 MOI (Figure 6A). AdUV killed H1299 lung cancer cells 64%, comparing with 8% of Adhz60 and 18% of Ad5 treatment at the same condition (0.3 MOI). Compared with the *E1b*-deleted Adhz60, AdUV also efficiently destroyed H441 lung cancer cells and MCF-7 breast cancer cells (Figure 6A). The EC50s of AdUV were 0.99, 1.12, 1.12, and 1.76 for A549, H1299, H441 and MCF-7, respectively (Figure 6B). Adjusted p-values indicated that statistically significant differences were observed between AdUV and Adhz60 treatment groups in all cancer cell lines treated with 1 MOI (Figure 6C). Therefore, AdUV induced the oncolysis of multiple cancer cell lines much more effectively than Adhz60.

## 4. Discussion

All clinical studies have shown that the efficacy of Ad virotherapy, specifically *dl*1520/ONYX-015 and H101, has remained low [22,24,30,31,42,43,44,45]. Reports have shown that viral spread within solid tumors was limited to cancer cells in the immediate vicinity of the injection site [46,47]. We recently reported that intratumorally injected Ads were localized within capsule-like structures which may have prevented virus spread throughout the entire tumor [16]. In a clinical study, oncolytic Ads were observed in clusters of 5–20 cells after intratumoral administration [48]. These data indicate that Ad spread is restricted in tumors and this restriction may significantly antagonize the efficacy of virotherapy [33].

The development of Ad vectors with increased spread would likely enhance the efficacy of virotherapy for the treatment of solid tumors. Increased Ad replication and cancer cell lysis are expected to enhance viral spread as more viruses are produced and then burst from infected cancer cells to facilitate Ad tumoral spread. Most Ads used in clinical studies have been subjected to genetic manipulation of the E1 genes. We have shown the deletion of the E1b genes repress the replicative potential of these Ads *in vitro* [35]. E1 modified Ads have been studied clinically because the functions of many other Ad genes and their effects upon Ad replication in cancer cells are still not completely understood. Therefore, it is difficult to predict the biological consequences following the genetic manipulation of other viral genes. An alternative strategy to develop oncolytic Ads is to use random mutagenesis to introduce changes to the viral genome and then select for Ads with the desired therapeutic properties. Such directed evolution has been shown to enhance the oncolytic efficacy of other viruses such as the recombinant Newcastle disease virus MTH87 [49] and Ad5 [50,51]. In this study, we treated Ad5 *dl*309 with UV irradiation and then selected AdUV from the pool of the UV-irradiated viruses for efficient replication in and release from cancer cells. This approach differs from previous reports as we treated Ad particles with DNA-damaging agent 72 times throughout the bioselection process. After 72 cycles of UV treatment and cancer selection, AdUV was isolated, which formed larger viral plaques than both Adhz60 and Ad5 in cancer cells, indicating greater viral spread. Furthermore, AdUV displayed greater anti-cancer potency and efficacy in all cancer cell lines tested. These studies have demonstrated the therapeutic efficacy of AdUV *in vitro*.

Autophagy is a regulated process of degradation and recycling of cellular constituents; the process is important in organelle turnover and the bioenergetics management of starvation [52,53]. During autophagy, the precursor form of LC3 is post-modified into two forms, LC3-I and LC3-II [39]. LC3-I is localized in the cytosol while LC3-II associated with autophagosomal membranes. LC3-II can be used to estimate the abundance of autophagosomes before they are destroyed through fusion with lysosomes [40]. Previously, we have shown that Ads induce autophagy by increasing the conversion of LC3-I to LC3-II that positively correlates with viral replication and oncolytic cell death and that autophagy may generate nutrients that can be used to support Ad replication [37]. In this report, we observed that AdUV induced autophagy more effectively than both Ad5 and Adhz60 in cancer cells. Verapamil treatment, an L-type Ca^2+^ channel antagonist which induces autophagy, has also been shown to increase Ad spread between cancer cells *in vitro* indicating the increased autophagic stimulation by AdUV may explain this increased spread phenotype [54]. Furthermore 3MA treatment, a phosphatidylinositol 3-kinase (PI-3K) and autophagosome formation inhibitor, was shown to inhibit the replication and oncolysis of Ad infected cancer cells [37]. Ad-mediated cancer cell lysis may also be related to the induction of apoptotic [17] and necrotic [55] cancer cell death pathways. Conflicting reports also indicate that wildtype serotype 5 Ads (Ad5) kills cancer cells independent of caspase activation and p53 expression [55,56]. While the molecular mechanism of enhanced AdUV cytolysis remains unclear, enhanced autophagy induction may play an important role.

**Figure 7:**
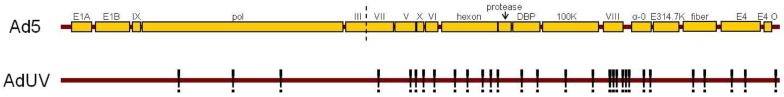
AdUV DNA mutation map. 30 mutations were detected in the AdUV genome in total, relative to the adenovirus serotype 5 (Ad5) reference sequences. No mutations were present in the early genes. The majority of these mutations were contained in adenovirus late genes. Mutations are indicated by exclamation points. Data was collected via ION torrent DNA sequencing and viewed with open source IGV software.

One limitation of this approach is that multiple mutations may be introduced into the viral genome. As expected, preliminary DNA analysis revealed several mutations in the AdUV genome (Figure 7). Most of these mutations were in the late genes encoding for viral structure proteins; however, no mutations were detected in the E1 region. Some of these mutations may directly affect the oncolytic properties of AdUV while others may have no direct effects. Therefore, ascertaining the effects of an individual mutation upon virotherapy from mutagenesis and cancer selection is complex. To determine which mutation or the combination of mutations have the greatest effect upon this enhanced oncolysis phenotype, additional *in vitro* and *in vivo* studies are necessary to analyze these mutations and is the subject of future investigation.

In this study, our results uncovered for the first time that AdUV, developed through the repeated mutagenesis and selection in cancer cells, has displayed many favorable characteristics and has great potential as a cancer treatment platform. The enhanced cancer cell lysis by AdUV appears to be related to the increased viral replication and release from infected cancer cells, as well as increased autophagy induction. This study has also indicated the utility of this approach for the development of other oncolytic viruses with designated therapeutic properties.

## Abbreviations

The following abbreviations are used in this manuscript:

Ad	Adenovirus
E1A	Early gene 1 A
E1B	Early gene 1 B
LC3	Microtuble-associated protein light chain 3
MOI	Multiplicity of infection
